# Levels of childhood vaccination coverage and the impact of maternal HIV status on child vaccination status in rural KwaZulu-Natal, South Africa*

**DOI:** 10.1111/j.1365-3156.2009.02382.x

**Published:** 2009-11

**Authors:** James Ndirangu, Till Bärnighausen, Frank Tanser, Khin Tint, Marie-Louise Newell

**Affiliations:** 1 Africa Centre for Health and Population Studies, University of KwaZulu NatalKwaZulu-Natal, South Africa; 2 The School of Public Health, University of the WitwatersrandWitwatersrand, South Africa; 3 Centre for Paediatric Epidemiology and Biostatistics, University College London Institute of Child HealthLondon, UK

**Keywords:** vaccination coverage, maternal HIV status, rural Africa

## Abstract

**Objectives:**

To analyse coverage of childhood vaccinations in a rural South African population and investigate whether maternal HIV status is associated with children’s vaccination status.

**Methods:**

2 431 children with complete information, 12–23 months of age at some point during the period January 2005 through December 2006 and resident in the Africa Centre Demographic Surveillance Area at the time of their birth were investigated. We examined the relationship between maternal HIV status and child vaccination status for five vaccinations [Bacillus Calmette-Guérin (BCG), diphtheria-tetanus-pertussis (DTP3), poliomyelitis (polio3), hepatitis B (HepB3), and measles] in multiple logistic regressions, controlling for household wealth, maternal age, maternal education and distances to roads, fixed and mobile clinics.

**Results:**

Coverage of the five vaccinations ranged from 89.3% (95% CI 81.7–93.9) for BCG to 77.3% (67.1–83.6) for measles. Multivariably, maternal HIV-positive status was significantly associated with lower adjusted odds ratios (AOR) of child vaccination for all vaccines [(AOR) 0.60–0.74, all *P*≤ 0.036] except measles (0.75, *P*= 0.073), distance to mobile clinic was negatively associated with vaccination status (all *P*≤ 0.029), household wealth was positively (all *P*≤ 0.013) and distance to nearest road negatively (all *P*≤ 0.004) associated with vaccination status.

**Conclusion:**

Positive maternal HIV status independently reduces children’s probability to receive child vaccinations, which likely contributes to the morbidity and mortality differential between children of HIV-positive and HIV-negative mothers. As a means of increasing vaccination coverage, policy makers should consider increasing the number of mobile clinics in this and similar communities in rural Africa.

## Background

Child vaccinations are among the most cost-effective public health interventions (Armstrong 2007). Globally, vaccinations have led to reduced morbidity (Ekanem *et al.* 2000; Edwards 2005; Ghendon *et al.* 2006) and mortality (Creese & Henderson 1980; Ekanem *et al.* 2000; Whitney & Pickering 2002) in children. Nevertheless, child vaccination coverage, especially in developing countries, is still far from universal, leading to preventable mortality. According to the World Health Organization (WHO), childhood vaccinations could have prevented an estimated 2.9 million deaths in children in 2007 (WHO-UNICEF 2007). In South Africa, in 2003 coverage of measles vaccination was 62% in children aged 12–23 months and thus fell far short of the 90% target for measles coverage set by the national Department of Health (Department of Health 2005); coverage with polio3 was 65% and thus fell short of the benchmark of 80% recommended by WHO to achieve polio eradication (WHO/AFRO 2003).

Factors that are determinants of childhood vaccination are maternal age (Breiman *et al.* 2004), maternal education (Streatfield *et al.* 1990; Madise & Matthews 1999; Anand & Bärnighausen 2007; Ibnouf *et al.* 2007; Munthali 2007), distance to health care facilities (Guerin 1998; Reichler *et al.* 1998; Buor 2003; Ndiritu *et al.* 2006) and household wealth (INDEPTH NETWORK; Defo 1996). It is unclear whether mothers’ HIV status is associated with children’s vaccination status. A study conducted in pregnant women attending Voluntary Counseling and Testing (VCT) in Rakai, Uganda, found that children born to HIV-infected mothers were two times less likely to be vaccinated in the first year of life than children born to HIV-negative mothers (Mast *et al.* 2006). However, the findings from this study may not apply to other populations. People self-select to attend VCT and thus commonly differ from the general population in many characteristics, including health-seeking behaviour and HIV status (Eley 2008).

The relationship between a mother’s HIV status and vaccination status of her children will be especially important for population child health in countries with high HIV prevalence, such as South Africa (Shisana *et al.* 2005). The risk of mortality is increased for all children born to HIV-positive mothers irrespective of their own HIV status (Sewankambo *et al.* 2000; Urassa *et al.* 2001; Nakiyingi *et al.* 2003; Newell *et al.* 2004). One reason for this increased mortality may be that children born to HIV-positive mothers are less likely to receive routine childhood vaccinations. HIV-positive mothers may be too weak or too poor to bring their children to vaccination clinics (Mast *et al.* 2006; Eley 2008) or they may be reluctant to access primary health care clinics for fear of stigma (Aggleton & Parker 2002). HIV infection may also be an indicator of relatively high tolerance of health risks in general, and mothers who are more tolerant of risks to their own health may be less motivated to ensure that their children receive risk-reducing interventions, such as vaccinations (Mast *et al.* 2004).

We use data from a large demographic surveillance in rural KwaZulu-Natal, South Africa to analyse coverage of childhood vaccinations and to investigate the relationship between maternal HIV status and child vaccination status.

## Methods

### Study area

The Africa Centre Demographic Surveillance Area (DSA), in Umkhanyakude district of northern KwaZulu-Natal, South Africa, covers 438 km^2^ and a total population of 87 000 (Muhwava & Nyirenda 2007) in 11 000 households enumerated twice a year since 2000 (Tanser *et al.* 2008, 2009). The area includes a formally designated urban township, peri-urban areas, and rural areas (Tanser *et al.* 2001). All homesteads in the study area have been mapped by fieldworkers using differential global positioning systems (GPS) and the homesteads database is continuously updates as new homesteads are built (Tanser *et al.* 2001).

The community has high HIV prevalence, approximating 40% in women attending antenatal clinics (Wilkinson *et al.* 1999; Rice *et al.* 2007). In the sub-district, the health service infrastructure comprises a central community hospital, 16 fixed clinics and 31 mobile clinic points (which are visited twice a month) (Tanser *et al.* 2001). The mobile clinics offer childhood vaccination in addition to family planning advice and antenatal care.

## Sample inclusion criteria

Our sample includes 2431 children who met the following criteria: 12–23 months of age at some point in time during the period of data collection from January 2005 through December 2006; resident in the Africa Centre Demographic Surveillance Area; their mothers were registered in the Africa Centre Demographic Information System (ACDIS) at the time of birth of the child.

## Survey methods

Trained interviewers visited households and administered a standardised questionnaire in the local language, isiZulu (Africa Centre for Health and Population Studies; Tanser *et al.* 2008). Women were asked to show the interviewers the South African Road-To-Health (RTH) card for all children aged 12–23 months at the time of the surveillance visit. The RTH card includes dates of all routine vaccinations a child has received (Tarwa & De Villiers 2007). When a child’s RTH card was missing, the interviewers asked the mother to recall whether her child had received each of the vaccinations included in the South African National Immunization schedule (Department of Health). This approach is similar to that used by the Demographic and Health Surveys (DHS) eliciting child vaccination status (Demographic and Health Surveys 2008). Langsten and Hill (1998) demonstrated in a study in Egypt that mothers’ recall can be an accurate source of children’s vaccination status.

For maternal HIV status, we used data from an annual population-based HIV surveillance in the Africa Centre DSA. All women aged 15–49 and men aged 15–54 years of age resident in the DSA were eligible for HIV testing in the surveillance. Trained field workers visited each eligible individual in his or her household. After written informed consent, the field worker collected blood by finger prick and prepared dried blood spots for HIV testing according to the Joint United Nations Programme on HIV/AIDS (UNAIDS) and the WHO guidelines (Africa Centre for Health and Population Studies; Bärnighausen *et al.* 2007; Tanser *et al.* 2008). The consent rate observed in the sample of mothers included in this study was about 50%, which is roughly similar to the rate observed in other studies in KwaZulu-Natal (Shisana *et al.* 2005; Welz *et al.* 2007). Possible reasons for non-consent to participation in the HIV surveillance include survey fatigue and knowledge of HIV status from voluntary testing and counseling in the public health services freely available to members of this community.

## Vaccination coverage analysis

We counted as vaccinated all children who had received all the doses of a specific vaccine as required by the South African National Immunization Schedule (Department of Health 1998): one vaccine dose Bacillus Calmette-Guérin (BCG) and measles, three doses in the case of diphtheria-tetanus-pertussis (DTP), poliomyelitis (polio), and Hepatitis B (HepB). The Immunization Schedule (Department of Health 1998) recommends the following ages for these vaccinations: at birth (BCG), at about 9 months of age (measles), at 6, 10 and 14 weeks of age (DTP1-3, polio1-3, and HepB1-3). Vaccination coverage was calculated as follows: the numerator was the number of children aged 12–23 months at the time of the survey who had received specified vaccine(s), at any time before the survey, according to information from a RTH card or report by the mother. The denominator was the total number of children aged 12–23 months at the time of the survey. This is a standard definition of vaccination coverage used, for instance, by the DHS (Demographic and Health Surveys 2008).

Aggregate levels of vaccination coverage can be potentially misleading as an indicator of programme effectiveness if there is substantial geographical heterogeneity in vaccination coverage within a population. We therefore sought to analyse the geographical variation in vaccination coverage across the study area to identify any low coverage communities for possible further intervention. We selected DTP3 coverage as a key coverage indicator because it is commonly used to monitor child health interventions (Murray *et al.* 2003; Murray & Stein 2009). To produce robust estimates of DTP3 coverage that vary across continuous geographical space we used a Gaussian kernel method (Waller & Gotway 2004) as described by Tanser *et al.* (2009). All 2 293 (94% of the sample) of children who had a valid homestead identifier were geolocated in a geographical information system to an accuracy of <2 m and subjected to a 3 km Gaussian kernel. The results were then used to subdivide the study area into areas of equal DTP3 vaccination coverage according to the WHO classification of levels of DTP3 coverage (WHO 2003).

## Statistical analysis

We used logistic regression analyses to assess associations with child vaccination status. All analyses were performed in stata (Version 10; Stata Corporation, College Station, TX, USA).

In multivariable regressions, we included several variables whose associations with child vaccination are well documented, namely, maternal education (Streatfield *et al.* 1990; Joshi 1994; Breiman *et al.* 2004; Torun & Bakirci 2006; Anand & Bärnighausen 2007), maternal age (Breiman *et al.* 2004), access to health facilities (Reichler *et al.* 1997; Muller *et al.* 1998) and household wealth (INDEPTH NETWORK; Cui & Gofin 2007). In order to capture access to health facilities, we used three distance variables (distance to mobile clinic, fixed clinic and nearest road) as continuous variables, measured in kilometers.

As mother’s HIV status, we took the result of each mother’s HIV test in the surveillance, taken closest in time to the date of birth of her child included in our sample. About half (52%) of the children sampled had mothers who had not received an HIV test within the Africa Centre HIV surveillance. To deal with this group of children missing maternal HIV, we included these children in our sample, assigning the variable “maternal HIV status” the value “unknown”. In addition, we ran separate regression analyses excluding children of mothers with missing HIV status.

We measured household wealth with a household wealth index. As shown by Morris *et al.* (2000), household wealth indices are valid proxies for wealth in health surveys in rural Africa. Following Filmer and Pritchett (Smith 2002), we used as wealth index the first principal component obtained in a principal component analysis of information on house ownership, water source, energy, toilet type, electricity and 27 household assets. We categorised households as used in a number of studies in poor provinces of South Africa (Gyekye & Akinboade 2003; van der Berg & Louw 2004), including studies investigating the effect of wealth on health (Blaauw & Penn-Kekana 2002; Booysen 2002; Bärnighausen *et al.* 2007).

## Results

Of the 2669 children resident in the Africa Centre DSA at birth and aged 12–23 months at the time of the interview, 151 (5.6%) had mothers who were not resident in the DSA, while for 87 (3.5%) information on distance to roads and clinics was missing. Our final sample for analysis thus included 2431 children, i.e. 91% of the total of 2669 children. A RTH card was available for 44% of the children included in our sample; for the remainder vaccination information was available from maternal recall. The median age of mothers was 24 years (IQR 13–54) with 11% HIV-positive, 37% HIV-negative and 52% of unknown HIV status (Table 1).

**Table 1:** Maternal and child characteristics of the study population

Variable	HIV-positive mothers (*n* = 275)	HIV-negative mothers (*n* = 890)	HIV status unknown (*n* = 1 266)	*P*-value
Wealth index				
Poorest	113 (41.1%)	383 (43.0%)	378 (29.9%)	<0.001
Medium	96 (34.9%)	347 (38.9%)	425 (33.6%)	
Wealthiest	50 (18.2%)	111 (12.5%)	277 (21.9%)	
Missing	16 (5.8%)	49 (5.6%)	186 (14.6%)	
Maternal age (years)				
<20	36 (13.1%)	290 (32.6%)	239 (18.9%)	<0.001
20–29	149 (54.2%)	367 (41.2%)	599 (47.3%)	
>=30	90 (32.7%)	233 (26.2%)	428 (33.8%)	
Maternal education (years)				
Primary education	74 (26.9%)	217 (24.4%)	255 (20.1%)	<0.001
Secondary education	167 (60.7%)	612 (68.8%)	761 (60.1%)	
Tertiary education and above	4 (1.5%)	13 (1.4%)	73 (5.8%)	
Missing	30 (10.9%)	48 (5.4%)	177 (14.0%)	
Child sex				
Female	135 (49.1%)	449 (50.5%)	631 (49.8%)	0.916
Male	140 (50.9%)	441 (49.5%)	635 (50.2%)	
Median distance to fixed clinic (IQR) km	2.6 (1.6–4.1)	3.1 (1.8–4.5)	2.8 (1.4–4.1)	0.002
Median distance to mobile Clinic (IQR) km	6.4 (3.3–8.7)	5.7 (3.4–7.7)	5.9 (3.2–8.2)	0.308
Median distance to primary road (IQR) km	1.6 (0.7–2.5)	1.7 (0.8–2.7)	1.5 (0.7–2.8)	0.031

Children were excluded from the analysis for one of two reasons: if they did not have information on the three geographic variables (distance to mobile clinic, fixed clinic and nearest road), or if their mothers were not resident in the DSA at the time of their birth. None of the children were excluded for both reasons. We compared the children who were included in the analysis to the children who were excluded because of the first reason and, separately, to the children excluded because of the second reason. No significant differences were found between any of the groups in maternal HIV status (*P*= 0.573), household wealth index (*P*= 0.915), distance to the nearest mobile clinic (*P*= 0.949), distance to the nearest road (*P*= 0.964), distance to the nearest mobile clinic (*P*= 0.949) and distance to the nearest road (*P*= 0.964). Compared to mothers who consented to an HIV test within the surveillance, mothers who did not consent were from wealthier households, had higher education levels and were older (Table 1).

**Figure 1:**
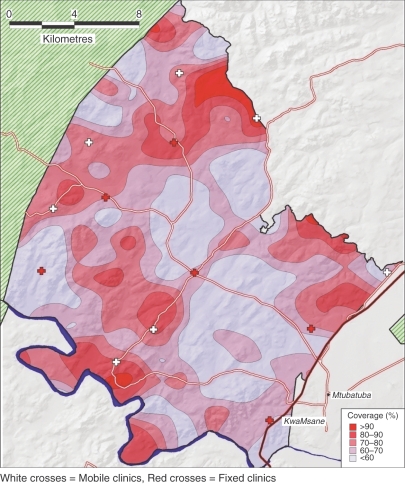
DTP3 vaccination coverage in rural KwaZulu-Natal.

Vaccination coverage was highest for BCG (89.3%, 95% confidence interval (CI) 81.7–93.9) and lowest for measles vaccine (77.3%, 95% CI 67.1–83.6). The other vaccination coverages were 87.3% for polio3 (95% CI 79.4–92.5), 84.9% for DTP3 (95% CI 76.6–90.6), and 81.7% for HepB3 (95% CI 73.0–88.1). There was marked geographic variation in DPT3 coverage ranging between 17.8% and 97.8% (overall mean = 84.1, 95% CI 75.7–89.9) (Figure 1).

In univariable analysis, there was evidence for an association between maternal HIV status and child vaccination status. The unadjusted odds ratios (OR) of maternal HIV status on individual vaccinations were 0.62 for BCG (95% CI: 0.41–0.94), 0.73 for polio3 (0.56–0.97), 0.73 for DTP3 (0.56–0.96), 0.74 for HepB3 (0.56–0.97) and 0.78 for measles (0.57–1.06) (Table 2). Living a closer distance to the mobile clinic was associated with higher odds of being vaccinated with BCG, polio3, DTP3 and HepB3. Relative household wealth was associated with higher odds of being vaccinated with measles but lower odds of being vaccinated with polio3, DTP3 and HepB3. Maternal age, maternal education, distance to fixed clinic and distance to nearest road were not associated with completed status for any of the vaccines (Table 2).

**Table 2:** Unadjusted odds of vaccination among children aged 12–23 months in rural KwaZulu-Natal

Variable	BCG OR 95% CI *P*-value	Polio3 OR 95% CI *P*-value	DTP3 OR 95% CI *P*-value	HepB3 OR 95% CI *P*-value	Measles OR 95% CI *P*-value
Maternal HIV status					
Negative	1.00	1.00	1.00	1.00	1.00
Unknown	0.74 (0.57–1.03)	0.84 (0.70–1.01)	0.85 (0.72–1.02)	0.84 (0.71–1.01)	0.88 (0.72–1.08)
	0.073	0.060	0.083	0.061	0.221
Positive	0.62 (0.41–0.94)	0.73 (0.56–0.97)	0.73 (0.56–0.96)	0.74 (0.56–0.97)	0.78 (0.57–1.06)
	0.024	0.028	0.027	0.029	0.111
Wealth index					
Poorest	1.00	1.00	1.00	1.00	1.00
Medium	0.93 (0.69–1.25)	0.78 (0.65–0.96)	0.79 (0.66–0.97)	0.80 (0.66–0.97)	1.12 (0.90–1.39)
	0.613	0.017	0.022	0.025	0.306
Wealthiest	1.28 (0.86–1.91)	0.76 (0.60–0.96)	0.77 (0.61–0.97)	0.78 (0.62–0.99)	1.35 (1.02–1.77)
	0.215	0.022	0.028	0.047	0.035
Missing	1.27 (0.78–2.06)	0.89 (0.66–1.19)	0.86 (0.65–1.16)	0.88 (0.66–1.18)	1.53 (1.08–2.18)
	0.337	0.433	0.329	0.408	0.017
Maternal age (years)					
<20	1.00	1.00	1.00	1.00	1.00
20–29	1.12 (0.81–1.55)	1.08 (0.88–1.33)	1.07 (0.87–1.32)	1.09 (0.88–1.34)	1.14 (0.90–1.45)
	0.488	0.468	0.493	0.429	0.263
>=30	1.10 (0.78–1.56)	1.05 (0.84–1.32)	0.98 (0.79–1.23)	1.03 (0.82–1.28)	1.04 (0.81–1.34)
	0.586	0.666	0.900	0.803	0.763
Maternal education (years)					
Primary education	1.00	1.00	1.00	1.00	1.00
Secondary education	0.89 (0.64–1.24)	0.97 (0.79–1.19)	0.97 (0.79–1.19)	1.01 (0.83–1.24)	1.16 (0.92–1.45)
	0.485	0.817	0.803	0.910	0.202
Tertiary education and above	0.93 (0.44–1.96)	1.03 (0.65–1.64)	1.01 (0.64–1.61)	0.99 (0.63–1.57)	1.22 (0.71–2.08)
	0.843	0.897	0.952	0.966	0.467
Missing	0.63 (0.39–0.99)	0.73 (0.54–0.98)	0.70 (0.52–0.95)	0.73 (0.54–0.99)	1.21 (0.85–1.72)
	0.044	0.038	0.022	0.042	0.289
Distance to mobile Clinic (km)	0.96 (0.92–0.99)	0.93 (0.91–0.96)	0.93 (0.90–0.95)	0.93 (0.90–0.96)	0.98 (0.95–1.01)
	0.045	<0.001	<0.001	<0.001	0.265
Distance to fixed Clinic (km)	1.04 (0.97–1.01)	1.05 (1.01–1.09)	1.05 (1.01–1.09)	1.04 (1.00–1.09)	1.00 (0.95–1.05)
	0.315	0.023	0.018	0.040	0.927
Distance to nearest road (km)	0.98 (0.93–1.05)	0.98 (0.94–1.02)	0.97 (0.94–1.01)	0.98 (0.94–1.02)	0.97 (0.93–1.01)
	0.661	0.261	0.217	0.285	0.122

OR, unadjusted odds ratio; CI, confidence interval.

When controlling for household wealth, maternal age, maternal education, distance to the nearest mobile clinic, distance to the nearest fixed clinic, and distance to the nearest road in multivariable analysis, children born to HIV-positive mothers had lower adjusted odds ratio (AOR) of having received the vaccinations than children of HIV-negative mothers: 0.60 for BCG (95% CI 0.39–0.92), 0.74 for polio3 (0.55–0.97), 0.74 for DTP3 (0.55–0.98), 0.74 for HepB3 (0.56–0.98) and 0.75 for measles (0.55–1.03) (Table 3). The size of the AOR for mothers with unknown HIV status ranked between the size of the AOR for HIV-positive and HIV-negative mothers. This ranking is expected since the group of mothers with unknown HIV status will include both HIV-positive and HIV-negative mothers. Holding all other variables constant, an increase in the distance to the nearest mobile clinic by 1 km decreased the odds of having received a vaccination by between 5% and 9% (all *P*≤ 0.029). Net of the other factors in the regression, children who live in households belonging to highest wealth tertile had significantly higher odds of having received BCG and measles vaccination than children living in poorer households [AOR 1.79 (1.12–2.84) and 1.59 (1.15–2.19), respectively] while an increase in distance to the nearest road showed evidence for an association with lower odds of receiving polio3, DTP3, and HepB3 vaccinations (6–7% reduction, all *P*≤ 0.004). All else being equal, maternal age, maternal education, and distance to fixed clinic showed no evidence for an association with child vaccination status.

**Table 3:** Adjusted odds of vaccination among children aged 12–23 months in rural KwaZulu-Natal

Variable	BCG AOR 95% CI *P*-value	Polio3 AOR 95% CI *P*-value	DTP3 AOR 95% CI *P*-value	HepB3 AOR 95% CI *P*-value	Measles AOR 95% CI *P*-value
Maternal HIV Status					
Negative	1.00	1.00	1.00	1.00	1.00
Unknown	0.72	0.85	0.86	0.85	0.80
	0.53–0.98	0.70–1.02	0.72–1.05	0.71–1.03	0.65–0.99
	0.037	0.089	0.144	0.090	0.044
Positive	0.60	0.74	0.74	0.74	0.75
	0.39–0.92	0.55–0.97	0.55–0.98	0.56–0.98	0.55–1.03
	0.019	0.034	0.036	0.036	0.073
Wealth index					
Poorest	1.00	1.00	1.00	1.00	1.00
Medium	1.08	0.88	0.91	0.90	1.21
	0.78–1.49	0.72–1.09	0.73–1.12	0.73–1.11	0.95-1.53
	0.642	0.249	0.355	0.334	0.117
Wealthiest	1.79	0.96	0.99	1.01	1.59
	1.12–2.84	0.73–1.27	0.76–1.32	0.77–1.34	1.15–2.19
	0.013	0.774	0.995	0.933	0.005
Missing	1.47	0.93	0.89	0.92	1.57
	0.89–2.46	0.68–1.26	0.66–1.22	0.68–1.25	1.09–2.26
	0.134	0.631	0.498	0.600	0.016
Maternal age (years)					
<20	1.00	1.00	1.00	1.00	1.00
20–29	1.20	1.12	1.11	1.12	1.18
	0.86–1.68	0.90–1.38	0.89–1.38	0.91–1.39	0.93–1.51
	0.276	0.312	0.337	0.286	0.173
>=30	1.13	1.08	1.00	1.06	1.09
	0.78–1.63	0.85–1.37	0.79–1.27	0.84–1.35	0.84–1.42
	0.521	0.529	0.997	0.605	0.515
Maternal education					
None	1.00	1.00	1.00	1.00	1.00
Primary education	0.83	0.97	0.94	0.99	1.07
	0.58–1.18	0.78–1.21	0.76–1.17	0.80–1.24	0.84–1.37
	0.297	0.802	0.593	0.974	0.556
At least secondary education	0.79	1.12	1.09	1.05	1.07
	0.37–1.72	0.69–1.82	0.68–1.77	0.65–1.70	0.62–1.86
	0.561	0.646	0.715	0.823	0.804
Missing	0.63	0.79	0.75	0.79	1.19
	0.39–1.02	0.58–1.08	0.55–1.03	0.57–1.08	0.83–1.73
	0.058	0.141	0.078	0.139	0.328
Distance to mobile Clinic (km)	0.93	0.92	0.91	0.91	0.95
	0.88–0.98	0.89–0.95	0.88–0.94	0.88–0.95	0.92–0.99
	0.018	<0.001	<0.001	<0.001	0.029
Distance to fixed clinic (km)	1.02	0.99	0.99	0.99	1.02
	0.94–1.11	0.95–1.05	0.95–1.05	0.94–1.04	0.96–1.08
	0.662	0.982	0.953	0.730	0.601
Distance to nearest road (km)	0.97	0.93	0.911	0.94	0.96
	0.91–1.03	0.89–0.98	0.88–0.94	0.89–0.98	0.91–1.00
	0.292	0.003	0.001	0.004	0.065
Sample size	2431	2431	2431	2431	2431
Log likelihood	−806.33	−1583.61	−1590.98	−1591.95	−1316.93
Likelihood ratio test - chi2	23.83	46.61	52.74	48.86	23.52
p-value chi2	0.0327	<0.0001	<0.0001	<0.0001	0.0358
Pseudo R2	0.0146	0.0145	0.0163	0.0151	0.0089

AOR, adjusted odds ratio; CI, confidence interval.

The main regression results are robust to the exclusion of individuals with mothers with unknown HIV status. None of the AOR changed by more than 5%. In particular, the AOR of maternal HIV status on individual vaccinations were 0.60 for BCG (95% CI 0.39–0.93), 0.72 for OPV3 (0.54–0.97), 0.72 for DTP3 (0.54-0.96), 0.72 for HepB3 (0.54-0.96) and 0.76 for measles (0.55–1.05). Further, the main results remained robust to the exclusion of individuals with missing data on wealth or maternal education. Particularly, the AOR of maternal HIV status on individual vaccinations were 0.54 for BCG (95% CI 0.34–0.85), 0.67 for OPV3 (0.49–0.92), 0.68 for DTP3 (0.50–0.92), 0.67 for HepB3 (0.49–0.92) and 0.71 for measles (0.51–0.99).

## Discussion

We have shown high coverage of childhood vaccination in the Africa Centre DSA ranging from 89.3% for BCG to 77.3% for measles with marked geographic variation of DTP3 in the population (mean ranging from 17.8% to 97.8%). The children born to HIV-positive mothers in the DSA are significantly less likely to receive routine childhood vaccinations in the first year of life than children born to HIV-negative mothers, when controlling for maternal age, household wealth, and distance to mobile clinics, fixed clinics and nearest road. The lower likelihood of vaccination coverage adds to other disadvantages children of HIV-positive mothers are likely to experience (Newell *et al.* 2004; Mast *et al.* 2006).

Several pathways can explain the relationship between mothers’ HIV status and children’s vaccination status. A mother who is HIV-positive will be more likely to suffer from disease and may be physically weaker than an HIV-negative mother (Keogh *et al.* 1994). She may thus find it more difficult to take her child to a vaccination clinic. HIV-related disease and weakness are likely to mediate of the effect of maternal HIV status on child vaccination status in this community, because walking is the most common mode of transport to health care clinics. About two-thirds of the population walk to clinics, and of those who walk 65% have to travel one hour or more to reach the nearest clinic (Tanser *et al.* 2006). HIV-positive mothers may also be less likely than HIV-negative mothers to bring their children to health care clinics to receive vaccinations because they have to spent time and resources accessing antiretroviral treatment (ART) and seeking care for HIV-associated diseases which are then not available to take their children to vaccination clinics.

In previous studies in developing countries, distance to primary health care facilities significantly predicted vaccination status (Muller *et al.* 1998; Torun & Bakirci 2006). We find that, *ceteris paribus*, distance to the nearest mobile clinic is significantly associated with child vaccination status, while distance to the nearest fixed clinic is not. The mobile clinics in this community provide only vaccinations (and some antenatal care) while the fixed clinics provide comprehensive primary care, of which vaccinations are only one small component. One reason why distance to the fixed clinics is not a significant predictor of vaccination status may thus be that mothers perceive mobile clinics to be the best places for their children to receive vaccinations and thus preferentially use mobile clinics for these vaccination services.

Studies from Africa have found a positive relationship between socio-economic status and vaccination status (INDEPTH NETWORK 2005; Ndiritu *et al.* 2006). In this study, we find that children belonging to the wealthiest households have improved vaccination outcomes for those routine childhood vaccines that are given only once (BCG and measles vaccinations) but that household socioeconomic status does not affect children’s probability to receive complete coverage with those vaccines that are given three times (polio3, DTP3 and HepB3). Children belonging to wealthier households may be more likely to have their vaccination coverage checked and to receive missing doses of vaccines when attending a health care facility than children of poorer households. While such a differential in quality of care by household socioeconomic status may significantly improve children’s likelihood to receive single-dose vaccinations, for the three-dose vaccinations the effect may be dominated by other factors determining child care (maternal HIV status, distance to the nearest mobile clinic, and distance to the nearest road).

In this largely rural area, mothers’ education and age were not significantly associated with vaccination status for any of the vaccinations. This finding is in contrast to other studies, which emphasize the importance of maternal education (Caldwell 1979; Streatfield *et al.* 1990; Joshi 1994; Munthali 2007) and maternal age (Breiman *et al.* 2004) on childhood vaccination status. One possible explanation of this finding may be the high attendance of antenatal care in this community estimated at 99% (Hoque *et al.* 2008). During antenatal care visits mothers receive information about the need to have their children vaccinated. The high antenatal care coverage may thus eliminate a knowledge advantage regarding prevention interventions for children that may be associated with maternal education in other communities. Future analysis need to test this hypothesis.

Our study has several possible limitations. First, the HIV status of a large proportion of mothers was missing. However, our main findings are robust to exclusion of individuals with mothers whose HIV status was not measured in the HIV surveillance, strengthening the confidence in our main result, i.e. that maternal HIV status is associated child vaccination status. Second, a large proportion of vaccination data was based on mother’s recall, which could suffer from biases (recall bias, if mothers report vaccinations their children have not received). This could over-estimate the true vaccination coverage. Third, we do not have information on children’s HIV status and thus cannot directly control for it in our analyses. However, a large proportion (35%) of HIV-positive children would have died before their first birthday (Newell *et al.* 2004) and thus been excluded from our sample. Lastly, the study was conducted in 2005 and 2006, i.e. at a time when ART coverage in this area was low (Herbst *et al.* 2009) – the relationship between HIV status and vaccination status could be different in an area with high ART coverage.

While overall vaccination coverage in this community is high, there is substantial need for improvement. For all vaccinations, we find geographical subareas with low vaccination coverage. For instance, while DTP3 coverage is overall high (at 85%), it falls below 60% in one third of the study area. At 77% overall measles coverage is below the level needed for herd immunity (which is estimated at 93–95%; Nokes & Swintom 1997). The findings from our study suggest that future interventions to improve vaccination coverage need to take into account the relationship between maternal HIV status and childhood vaccination status. At the community level, health policy makers should consider intensified vaccination efforts in high HIV-prevalence areas. At the individual level, interventions targeting HIV-positive mothers and their children should incorporate vaccination promotion.
